# Impact of Long-Term Erythromycin Therapy on the Oropharyngeal Microbiome and Resistance Gene Reservoir in Non-Cystic Fibrosis Bronchiectasis

**DOI:** 10.1128/mSphere.00103-18

**Published:** 2018-04-18

**Authors:** Jocelyn M. Choo, Guy C. J. Abell, Rachel Thomson, Lucy Morgan, Grant Waterer, David L. Gordon, Steven L. Taylor, Lex E. X. Leong, Steve L. Wesselingh, Lucy D. Burr, Geraint B. Rogers

**Affiliations:** a Infection and Immunity, South Australia Health and Medical Research Institute, Adelaide, South Australia, Australia; b SAHMRI Microbiome Research Laboratory, Flinders University School of Medicine, Adelaide, South Australia, Australia; c Gallipoli Medical Research Centre, University of Queensland, Greenslopes Private Hospital, Brisbane, Australia; d Department of Respiratory Medicine, Concord Hospital Clinical School, University of Sydney, Sydney, New South Wales, Australia; e School of Medicine and Pharmacology Royal Perth Hospital Unit, The University of Western Australia, Perth, Australia; f Department of Microbiology and Infectious Diseases, Flinders University, Adelaide, South Australia, Australia; g Immunity, Infection and Inflammation Program, Mater Research Institute, University of Queensland, Translational Research Institute, Woolloongabba, Queensland, Australia; h Department of Respiratory Medicine, Mater Health Services, South Brisbane, Queensland, Australia; JMI Laboratories

**Keywords:** antibiotic resistance, bronchiectasis, macrolide therapy, oropharyngeal microbiome

## Abstract

Long-term macrolide therapy reduces rates of pulmonary exacerbation in bronchiectasis. However, little is known about the potential for macrolide therapy to alter the composition and function of the oropharyngeal commensal microbiota or to increase the carriage of transmissible antimicrobial resistance. We assessed the effect of long-term erythromycin on oropharyngeal microbiota composition and the carriage of transmissible macrolide resistance genes in 84 adults with bronchiectasis, enrolled in the Bronchiectasis and Low-dose Erythromycin Study (BLESS) 48-week placebo-controlled trial of twice-daily erythromycin ethylsuccinate (400 mg). Oropharyngeal microbiota composition and macrolide resistance gene carriage were determined by 16S rRNA gene amplicon sequencing and quantitative PCR, respectively. Long-term erythromycin treatment was associated with a significant increase in the relative abundance of oropharyngeal Haemophilus parainfluenzae (*P* = 0.041) and with significant decreases in the relative abundances of Streptococcus pseudopneumoniae (*P* = 0.024) and Actinomyces odontolyticus (*P* = 0.027). Validation of the sequencing results by quantitative PCR confirmed a significant decrease in the abundance of *Actinomyces* spp. (*P* = 0.046). Erythromycin treatment did not result in a significant increase in the number of subjects who carried *erm*(A),* erm*(B), *erm*(C), *erm*(F),* mef*(A/E), and *msrA* macrolide resistance genes. However, the abundance of *erm*(B) and *mef*(A/E) gene copies within carriers who had received erythromycin increased significantly (*P* < 0.05). Our findings indicate that changes in oropharyngeal microbiota composition resulting from long-term erythromycin treatment are modest and are limited to a discrete group of taxa. Associated increases in levels of transmissible antibiotic resistance genes within the oropharyngeal microbiota highlight the potential for this microbial system to act as a reservoir for resistance.

**IMPORTANCE** Recent demonstrations that long-term macrolide therapy can prevent exacerbations in chronic airways diseases have led to a dramatic increase in their use. However, little is known about the wider, potentially adverse impacts of these treatments. Substantial disruption of the upper airway commensal microbiota might reduce its contribution to host defense and local immune regulation, while increases in macrolide resistance carriage would represent a serious public health concern. Using samples from a randomized controlled trial, we show that low-dose erythromycin given over 48 weeks influences the composition of the oropharyngeal commensal microbiota. We report that macrolide therapy is associated with significant changes in the relative abundances of members of the *Actinomyces* genus and with significant increases in the carriage of transmissible macrolide resistance. Determining the clinical significance of these changes, relative to treatment benefit, now represents a research priority.

## Abstract (precis)

Recent demonstrations that long-term macrolide therapy can prevent exacerbations in chronic airways diseases have led to a dramatic increase in their use. However, little is known about the wider, potentially adverse impacts of these treatments. Substantial disruption of the upper airway commensal microbiota might reduce its contribution to host defense and local immune regulation, while increases in macrolide resistance carriage would represent a serious public health concern. Using samples from a randomized controlled trial, we show that low-dose erythromycin given over 48 weeks influences the composition of the oropharyngeal commensal microbiota. We report that macrolide therapy is associated with significant changes in the relative abundances of members of the *Actinomyces* genus and with significant increases in the carriage of transmissible macrolide resistance. Determining the clinical significance of these changes, relative to treatment benefit, now represents a research priority.

## INTRODUCTION

Non-cystic fibrosis (non-CF) bronchiectasis is characterized by chronic infection and inflammation of the airways, which contributes to progressive lung damage (1). Long-term low-dose macrolide therapy has been shown to reduce the frequency of pulmonary exacerbations in patients with bronchiectasis (2–5). Similar beneficial effects have also been reported in cases of chronic obstructive pulmonary disease (6), cystic fibrosis (7), and severe asthma (8), leading to a substantial increase in the use of macrolides in chronic respiratory conditions (4).

Increased macrolide use will almost certainly result in substantial increases in the carriage of macrolide-resistant bacteria (9). However, the emergence of macrolide resistance is unlikely to result in a loss of efficacy in the treatment of chronic respiratory diseases, with no evidence that the ability of macrolides to reduce exacerbation rates diminishes with increasing resistance gene carriage in potential respiratory pathogens, including Haemophilus influenzae, Streptococcus pneumoniae, Staphylococcus aureus, and Moraxella catarrhalis (3). The absence of a close association between resistance and treatment efficacy is likely to reflect the hypothesized mode of action of macrolides in chronic respiratory diseases, which is principally immunomodulatory rather than bactericidal or bacteriostatic (10, 11). However, prolonged macrolide treatment might yet have deleterious consequences through its impact on commensal bacterial communities, including communities in the upper respiratory tract.

The close resemblance between microbiota of the oropharynx and microbiota of healthy lungs suggests frequent dispersal of bacteria between these sites (12). The presence of a commensal microbiota in the oropharynx reduces infection susceptibility, both through the regulation of local immunity (13) and the competitive exclusion of pathogens (14). Disruption of the oropharyngeal microbiota, for example, as a result of antibiotic exposure, can result in the overgrowth of potential pathogens and an increased risk of associated local or disseminated respiratory infections (15). In addition, while the emergence of macrolide resistance is not a major concern in regard to the treatment of patients with chronic respiratory diseases, the potential transmission of resistant pathogens to vulnerable individuals (16), where they can be the cause of serious acute infections (17), must be considered.

Evidence that macrolide therapy is likely to have an impact on oropharyngeal microbiology was provided by the four randomized controlled trials performed in bronchiectasis patients to date; all four trials have resulted in reports of increased levels of macrolide-resistant streptococci (2–5). Macrolide resistance can also develop in nonstreptococcal pathogens, including Legionella pneumophila (18), Bordetella pertussis (19), the highly pathogenic Mycobacterium abscessus (20), and S. aureus (21), and can persist long after treatment has ceased (21). Furthermore, the majority of macrolide resistance determinants are encoded on mobile genetic elements, meaning that there is potential for horizontal transmission between commensal and pathogen populations (22).

Beyond the selection of macrolide-resistant streptococci, the impact of long-term macrolide exposure on the oropharyngeal microbiota is largely unknown. Currently, little data exist regarding resultant changes in either the relative abundance of taxa or the carriage of resistance determinants. To address this important knowledge gap, we assessed the impact of 12 months of low-dose erythromycin on oropharyngeal microbiota composition and antibiotic resistance gene carriage in participants with non-CF bronchiectasis in the Bronchiectasis and Low-dose Erythromycin Study (BLESS).

## RESULTS

### Oropharyngeal microbial community in individuals with bronchiectasis.

Oropharyngeal microbiota compositions at baseline (see Fig. S1 in the supplemental material) did not differ significantly between the erythromycin and control groups (analysis of similarity [ANOSIM] *R* = 0.021, *P* = 0.072; Fig. S2A). Since intergroup differences in the use of inhaled short-acting β-agonists (SABA) were observed, the baseline oropharyngeal microbiota compositions determined for patients with or without SABA use were compared. No significant compositional differences were observed between patients with or without SABA use (ANOSIM *R* = −0.008, *P* = 0.602), suggesting that differences in SABA use do not have a major influence on the intergroup microbiota analysis.

10.1128/mSphere.00103-18.2 FIG S1  Oropharyngeal microbiota composition of participants in the erythromycin or placebo treatment group at baseline and 48 weeks after the commencement of treatment. Download FIG S1, TIF file, 1.1 MB. © Crown copyright 2018. 2018 Crown This content is distributed under the terms of the Creative Commons Attribution 4.0 International license.

10.1128/mSphere.00103-18.3 FIG S2  Nonmetric multidimensional scaling (NMDS) ordination plots of erythromycin (blue) and placebo (red) patients at baseline and 48 weeks after the commencement of treatment. Statistical analysis to determine the significance of microbiota composition differences between treatment groups was performed using the ANOSIM test (*P* < 0.05). Download FIG S2, TIF file, 0.2 MB. © Crown copyright 2018. 2018 Crown This content is distributed under the terms of the Creative Commons Attribution 4.0 International license.

**FIG 1:**
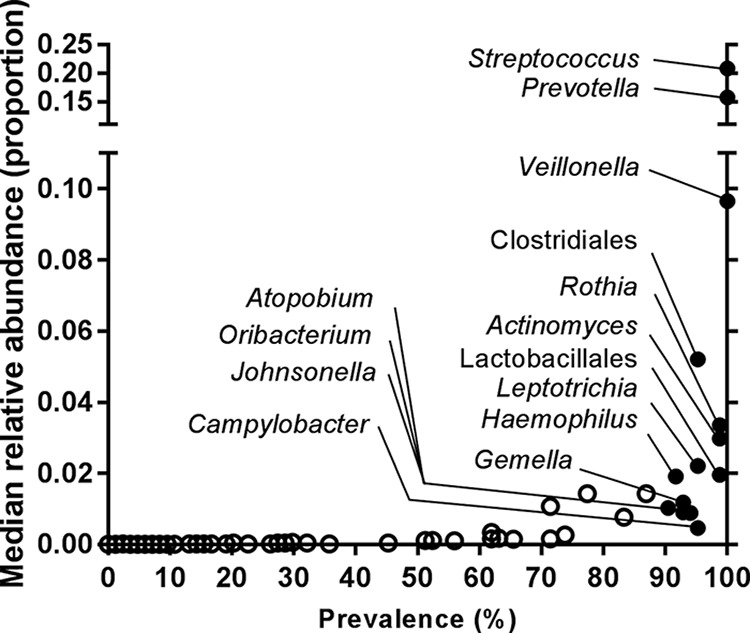
Members of the core oropharyngeal microbiota at baseline, identified based on a relative abundance of ≥0.1% within subjects in at least 90% of the study population.

**FIG 2:**
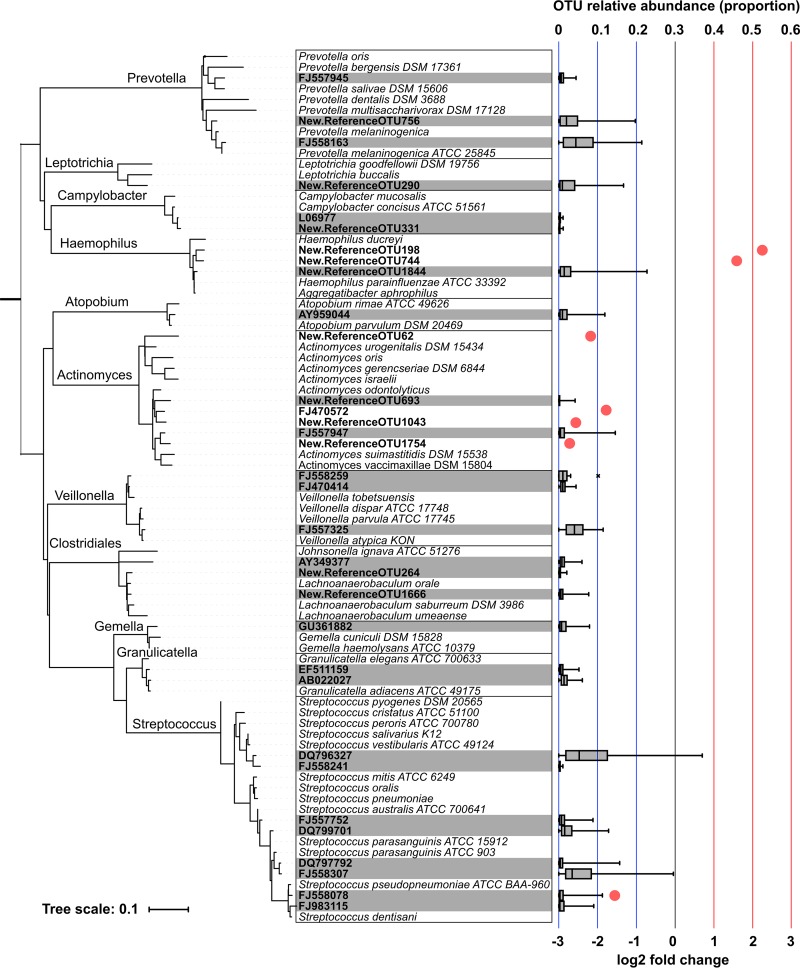
Phylogenetic relationship of OTUs comprising the core microbiota and OTUs that contributed to the microbial community differences between the placebo-treated and erythromycin-treated groups after 48 weeks of low-dose erythromycin. The relative abundances of OTUs that constituted the core microbiota are represented by the horizontal boxplots. The OTUs that significantly differed between the placebo and erythromycin groups after 48 weeks of treatment are indicated, with the log_2_ fold changes represented by the red circles (FDR-adjusted *P* < 0.05).

At baseline, the core oropharyngeal microbiota (defined as the taxa found at a relative abundance of >0.1 in at least 90% of patients) was comprised of common oropharyngeal bacterial genera, including *Streptococcus*, *Prevotella*, *Veillonella*, *Rothia*, *Actinomyces*, *Leptotrichia*, *Haemophilus*, *Gemella*, *Oribacterium*, and *Campylobacter*, in addition to less extensively characterized taxa such as *Atopobium* and *Johnsonella* (taxon distributions are shown in Fig. 1) (Table 1). *Pseudomonas* and *Achromobacter*, genera that are commonly associated with poor lung disease outcomes, were observed at relative abundances of >0.1% in 12 and 2 patients, respectively. Core taxa represented multiple distinct operational taxonomic units (OTUs) within the *Bacteroidetes*, *Firmicutes*, *Proteobacteria*, *Fusobacteria*, and *Actinobacteria* phyla, which differed substantially in relative abundance between subjects (Fig. 2).

**TABLE 1:** Relative abundances of bacterial members of the core microbiota at baseline

Bacterial taxon	Median % cumulative relative abundance (IQR)
*Streptococcus*	20.8 (17.3–28.1)
*Prevotella*	15.8 (9.9–21.5)
*Veillonella*	9.7 (6.3–12.6)
*Actinomyces*	3.3 (1.9–6.8)
*Rothia*	3.0 (1.7–5.9)
*Leptotrichia*	2.2 (0.7–5.8)
*Haemophilus*	1.9 (0.6–4.3)
*Gemella*	1.2 (0.6–3.1)
*Atopobium*	1.0 (0.4–2.2)
*Oribacterium*	0.9 (0.4–1.8)
*Johnsonella*	0.9 (0.4–2.1)
*Campylobacter*	0.4 (0.3–1.0)
*Clostridiales*	5.2 (2.0–12.8)
*Lactobacillales*	2.0 (1.0–3.6)

### Impact of erythromycin treatment on the abundance of discrete bacterial taxa within the oropharynx.

Oropharyngeal microbiota compositions differed significantly between the erythromycin and placebo groups at 48 weeks (ANOSIM *R* = 0.054, *P* = 0.003) (Fig. S2B). Alpha diversity measures of microbial community evenness significantly decreased with time in both the erythromycin and placebo groups. However, pairwise comparison at each time point indicated no significant differences in other alpha diversity measures assessed (Fig. S3). There was also no difference in between-group or within-group total bacterial loads (placebo baseline load, 1.67 [median; interquartile range {IQR}, 0.40 to 3.60] × 10^8^ copies 16S rRNA/swab; placebo load at 48 weeks, 1.33 [0.41 to 3.20] × 10^8^; erythromycin baseline load, 1.66 [0.63 to 3.30] × 10^8^ copies 16S rRNA/swab; erythromycin load at 48 weeks, 1.80 [0.66 to 2.75] × 10^8^). However, significant changes were observed in the relative abundances of the members of a specific subset of bacterial taxa.

10.1128/mSphere.00103-18.4 FIG S3  Alpha diversity of microbial community richness, evenness, and diversity based on (A) observed species, (B) Simpson index (1-D), and (C) Shannon diversity or (D) Faith’s phylogenetic data of participants in the placebo group or the erythromycin group, respectively, at baseline and after 48 weeks. Pairwise statistical comparisons within groups across time and between groups at each time point were performed using the Wilcoxon test and Mann-Whitney test, respectively, at a significance level of 0.05. Download FIG S3, TIF file, 0.7 MB. © Crown copyright 2018. 2018 Crown This content is distributed under the terms of the Creative Commons Attribution 4.0 International license.

Fold change comparisons at the genus level between the erythromycin and placebo groups at 48 weeks performed using DESEQ2 software indicated decreased relative abundances of four OTUs assigned to *Actinomyces* in patients who received erythromycin (for OTU62, log_2_ fold change = −2.06 ± 0.53, false-discovery-rate [FDR]-adjusted *P* = 0.024; for OTU1043, log_2_ fold change = −2.51 ± 0.69, FDR *P* = 0.027; for OTU1754, log_2_ fold change = −2.69 ± 0.75, FDR *P* = 0.027; for FJ470572, log_2_ fold change = −1.86 ± 0.55, FDR *P* = 0.041) (Fig. 2). One OTU (FJ558078) assigned to the genus *Streptococcus* decreased in relative abundance (log_2_ fold change = −1.57 ± 0.41, FDR *P* = 0.024) whereas two OTUs assigned as *Haemophilus* increased in relative abundance in patients who received erythromycin (for OTU198, log_2_ fold change = 2.16 ± 0.61, FDR *P* = 0.027; for OTU744, log_2_ fold change = 1.65 ± 0.49, FDR *P* = 0.041) (Fig. 2). Two of the OTUs could be identified using the Ribosomal Database project (RDP) classifier tool, one as Actinomyces odontolyticus (RDP sequence match score of ≥90%) and the other as Haemophilus parainfluenzae (RDP sequence match score of 92%), consistent with their assignment within the bacterial phylogenetic tree. The *Streptococcus-*assigned OTU was phylogenetically closest to S. pseudopneumoniae (Fig. 2).

**FIG 3:**
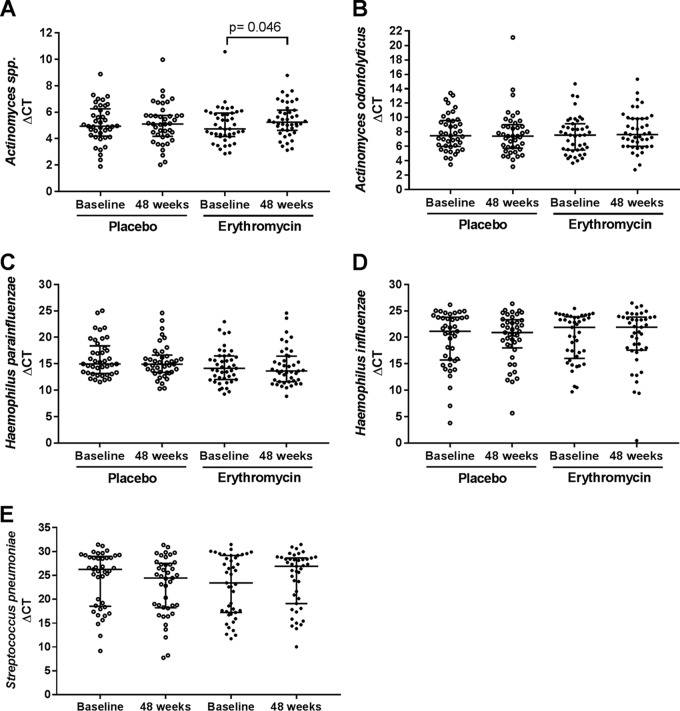
Comparison of Δ*C*_*T*_ values between the placebo group and erythromycin group at baseline and at 48 weeks for taxa that contributed to the differences between groups. (A) *Actinomyces* spp. (B) Actinomyces odontolyticus. (C) Haemophilus parainfluenzae. (D) Haemophilus influenzae. (E) Streptococcus pneumoniae or Streptococcus pseudopneumoniae. Statistical analyses of comparisons between paired samples from the placebo group and the erythromycin group were performed using the Wilcoxon test at a significance level of 0.05. A one-tailed test was used for the bacterial taxa *Actinomyces*, Actinomyces odontolyticus, and Haemophilus parainfluenzae, the relative abundances of which were identified by DESEQ2 analysis to be significantly altered.

Pairwise comparison of the relative abundances of these taxa within subjects at baseline and week 48 showed a consistent decrease in the relative abundance of *Actinomyces* OTUs in subjects receiving erythromycin but not in those receiving placebo (Fig. S4A to D). However, such consistent trends were not observed for H. parainfluenzae or S. pseudopneumoniae OTUs (Fig. S4E to G). Quantitative PCR, which was performed to validate the observed alterations in the relative abundances of discriminant taxa, supported these findings. Levels of the *Actinomyces* genus decreased significantly with erythromycin treatment (baseline Δ*C*_*T*_ = 4.73 [median; Δ*C*_*T*_ values are based on differences in threshold cycle {*C*_*T*_} values between the target gene and the reference {16S rRNA} gene], IQR = 4.17 to 5.94; Δ*C*_*T*_ at 48 weeks = 5.24, IQR = 4.63 to 6.15 [Wilcoxon test, one-tailed, *P* = 0.046]) but not placebo treatment (baseline Δ*C*_*T*_ = 4.94 [median], IQR = 4.33 to 6.25; Δ*C*_*T*_ at 48 weeks = 5.11, 4.18 to 5.75 [Wilcoxon test, one-tailed, *P* = 0.480]) (Fig. 3). However, treatment-associated differences in the levels of A. odontolyticus, H. parainfluenzae, and S. pseudopneumoniae did not achieve statistical significance (Fig. 3). In addition, quantitative PCR analysis of potentially pathogenic members of discriminant genera, including H. influenzae (placebo *P* = 0.758; erythromycin *P* = 0.513 [Wilcoxon test, two-tailed]) and S. pneumoniae (placebo *P* = 0.193; erythromycin *P* = 0.353 [Wilcoxon test, two-tailed]), indicated no significant difference in their absolute levels between the treatment and placebo groups.

10.1128/mSphere.00103-18.5 FIG S4  Pairwise comparisons of the relative abundances of OTUs that were significantly altered within subjects in the placebo and erythromycin groups at baseline and after 48 weeks. (A) *Actinomyces* spp. (OTU62). (B) Actinomyces odontolyticus (OTU1043). (C) *Actinomyces* spp. (FJ470572). (D) *Actinomyces* spp. (OTU1754). (E) Haemophilus parainfluenzae (OTU198). (F) Haemophilus parainfluenzae (OTU744). (G) Streptococcus pneumoniae (FJ558078). Download FIG S4, TIF file, 0.4 MB. © Crown copyright 2018. 2018 Crown This content is distributed under the terms of the Creative Commons Attribution 4.0 International license.

### Low-dose erythromycin significantly increases antibiotic resistance gene carriage within the oropharyngeal microbiota.

**FIG 4:**
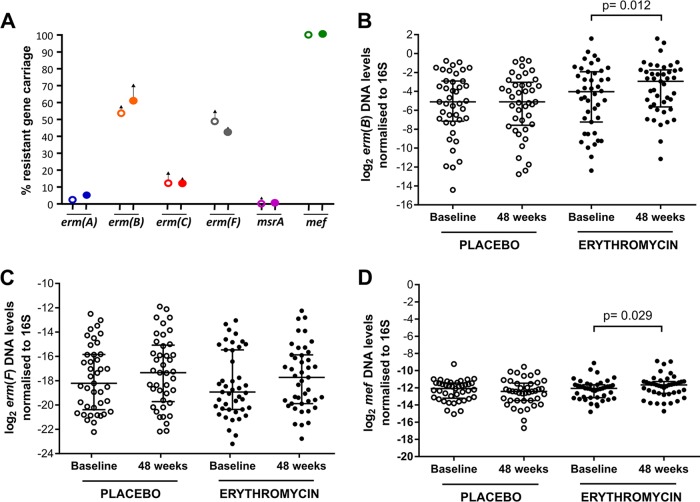
(A) Effects of erythromycin treatment on the carriage of antibiotic resistance genes, (B to D) Effects of erythromycin treatment on the levels of (B) *erm*(B), (C) *erm*(F), and (D) *mef* genes. The percentages of increase or decrease in antibiotic resistance gene carriage in the placebo group (open circle) and the erythromycin group (closed circle) at trial week 48 were calculated based on the increment or decrement from baseline values. Quantitative levels of the *erm*(B) and *mef* genes were normalized to the total bacterial load. The log_2_ DNA levels for the placebo and erythromycin groups at baseline and at the end of placebo or erythromycin treatment were plotted. Statistical analyses of differences between data from the different time points were performed using the Wilcoxon test at a significance level of 0.05.

*erm*(A), *erm*(B), *erm*(C), *erm*(F), *msrA*, and *mef* are transmissible macrolide resistance genes that are known to be carried by bacteria commonly found in the oropharynx (23–25). The carriage of these genes was assessed in the study population. At baseline, the most commonly carried resistance gene was *mef* (detected in most subjects), while *erm*(B) (placebo = 53.6%, erythromycin = 60.5%) and *erm*(F) (placebo = 53.6%, erythromycin = 41.9%) were detected in approximately half of the subjects (Table 2). Lower rates of carriage were observed for *erm*(C) (placebo = 17.1%, erythromycin = 11.6%) and *erm*(A) (placebo = 2.4%, erythromycin = 4.7%). *msrA* was detected in one subject at baseline. Neither the treatment group nor the control group showed a significant change in the rates of resistance gene carriage during the 48-week trial (Fisher's exact test, *P* > 0.05) (Fig. 4A) (Table 2).

**TABLE 2:** Antibiotic resistance gene carriage in the placebo and erythromycin groups at baseline and at the end of erythromycin treatment (48 weeks)

Resistance gene	% gene carriage in indicated group				*P* value (Fisher's exact test, 48 wks)
	Placebo		Erythromycin		
	Baseline	48 wks	Baseline	48 wks	
*erm*(A)	2.4	2.4	4.7	4.7	>0.99
*erm*(B)	53.6	56.1	60.5	69.8	0.26
*erm*(C)	17.1	12.2	11.6	14.0	>0.99
*erm*(F)	53.6	48.8	41.9	44.2	0.82
*msrA*	0.0	2.4	0.0	0.0	0.49
*mef*	100.0	95.1	100.0	97.7	0.61

As the *erm*(B), *erm*(F), and *mef* resistance genes were found to be common within the study population, levels of resistance gene carriage within subjects were assessed by quantifying the resistance gene copy number and were normalized to total bacterial load. Levels of both *erm*(B) and *mef* increased significantly between baseline and week 48 in the treatment group [*erm*(B) baseline, median log_2_ *erm*(B)/16S ratio = −4.03, IQR = −7.24 to −1.92; *erm*(B) at 48 weeks, median log_2_ *erm*(B)/16S ratio = −2.93, IQR = −5.65 to −1.73 [Wilcoxon test, *P* = 0.012]; *mef* baseline, median log_2_ *mef*/16S ratio = −12.07, IQR = −13.15 to −11.65; *mef* at 48 weeks, median log_2_ *mef*/16S ratio = −11.67, IQR = −12.64 to −11.27 [*P* = 0.029]) but not in the placebo group (Fig. 4B and D). In contrast, levels of *erm*(F) were not significantly altered in the control group or the treatment group after 48 weeks (Fig. 4C).

The relationship of PCR-based resistance gene detection to culture-based assessments of macrolide-resistant streptococci performed within the original trial (3) indicated that while 15 of the subjects assessed had no resistant streptococci isolated by culture, 8 were found to carry *erm*(B) and 1 carried the *erm*(C) resistance gene.

## DISCUSSION

Culture-based studies have clearly demonstrated that low-dose macrolide therapy exerts a substantial selective pressure in the oropharyngeal microbiota, as reflected in a proportional increase in the level of macrolide-resistant streptococci (3–5). Indeed, previous culture-based assessment of streptococci isolated from the subjects examined here showed that erythromycin resistance increased significantly during the trial period (median change, 27.7%, *P* = 0.001) (4). However, in contrast to the widespread disruption of human commensal microbiota that can result from acute antibiotic exposure (26), the changes in microbiota composition that were observed with erythromycin treatment were very modest and limited to a discrete group of taxa within the *Actinomyces* genus.

Our findings are broadly consistent with a previous study that assessed the impact of 6 months of azithromycin treatment in patients with severe asthma. Using 16S rRNA gene amplicon sequencing, Lopes et al. reported changes in oropharyngeal microbiota in a relatively small number of bacterial taxa, including members of the *Actinomyces* genus (27). The modest changes in microbiota composition that we found following long-term erythromycin treatment are likely to reflect a combination of the relatively weak selective pressure that low-dose erythromycin treatment represents and "microbiota resistance," a phenomenon by which complex bacterial systems can remain relatively unchanged in spite of the presence of a disruptive force (28). Microbiota resistance has been described in both the saliva of healthy individuals and the lower airways of patients with cystic fibrosis during antibiotic challenge (29, 30).

The observed decreases in *Actinomyces* abundance are consistent with the low relative tolerance of members of this genus with respect to macrolide antibiotics (31). Whether these reductions in *Actinomyces* abundance are of clinical significance, however, is not yet clear. Members of *Actinomyces*, a genus of Gram-positive, facultative anaerobes, are typically considered commensals and can be commonly detected in the oropharynx, gastrointestinal tract, and female genital tract of healthy individuals (31). However, *Actinomyces* species are capable of causing opportunistic lower respiratory tract infection, particularly in the form of pulmonary actinomycosis (31), including in patients with bronchiectasis (32, 33). While actinomycosis is most commonly associated with Actinomyces israelii (31), infections caused by A. odontolyticus, a species that was observed to be reduced in relative abundance with erythromycin treatment, have been reported in rare instances, including in pulmonary infections (34, 35).

Long-term antibiotic exposure is associated with the development of resistance that can persist well beyond the treatment period (21). Importantly, selection of resistance can occur even where the antibiotic concentration is below the MIC for a given bacterial population (36). We assessed carriage of six transmissible macrolide resistance genes that can be carried by common members of the oropharyngeal microbiota (24, 37, 38), observing a significant increase in the levels of *erm*(B) and *mef* genes. This finding is consistent with the increased carriage of *erm*(B) in streptococci reported in healthy individuals following 180 days of treatment with azithromycin or clarithromycin (39) and with culture-based assessments of resistance carriage following long-term macrolide use in bronchiectasis patients (40). While the *erm*(B) and *mef* genes are often associated with streptococcal pathogens (41), they are also common in nonstreptococcal respiratory pathogens, including H. influenzae (24) and S. aureus (42), and in upper respiratory tract commensals such as *Gemella* (22). Importantly, *erm*(B) and *mef* genes can move horizontally between bacterial species via conjugation or transformation (22, 24), allowing commensal taxa to act as resistance reservoirs.

The BLESS trial reported that subjects who received erythromycin treatment experienced significantly fewer pulmonary exacerbations than those who received placebo (4). In our study population, members of the erythromycin group also had a significant reduction in the number of exacerbations (*P* = 0.03), in keeping with prior trial findings. The fact that nonmacrolide antibiotic exposure was higher in the placebo group, but that no significant difference was observed between the placebo baseline microbiota composition and the week 48 composition, suggests that antibiotic therapy used for the treatment of pulmonary exacerbations did not contribute substantially to observed shifts in oropharyngeal microbiology.

Our study had a number of limitations that should be considered. Oropharyngeal swabs were available for only 84 of the 112 subjects (total *n* = 117) who completed the original BLESS trial, although the patient characteristics of the two cohorts were broadly comparable (see Table S1 in the supplemental material). Basing our analysis on subjects of the BLESS trial provided substantial advantages in terms of the uniformity of samples and the availability of patient metadata; however, the use of specific selection criteria for subjects means that our study population might not be representative of the wider bronchiectasis patient population. Furthermore, our analysis focused on the oropharynx, and while the impact of long-term erythromycin treatment on microbiota within other regions of the upper respiratory tract is likely to be consistent with the results reported here, changes in composition and resistome characteristics in other commensal populations must also be considered. Finally, our focus was on the detection of resistance gene carriage, and we did not assess whether these genes were expressed *in vitro*.

10.1128/mSphere.00103-18.6 TABLE S1  Comparison of characteristics of the current BLESS study population and those of the original cohort. Download TABLE S1, PDF file, 0.1 MB. © Crown copyright 2018. 2018 Crown This content is distributed under the terms of the Creative Commons Attribution 4.0 International license.

In summary, we report long-term erythromycin treatment in adult patients with bronchiectasis to be associated with limited changes in the composition of the oropharyngeal microbiota, confined to members of the genus *Actinomyces*. These changes were, however, modest and limited to shifts in the relative abundances of a discrete group of bacterial species. Significant increases in within-subject levels of some transmissible macrolide resistance genes highlight the potential for the oropharynx to act as a reservoir for antimicrobial resistance.

## MATERIALS AND METHODS

### Study design and setting.

A detailed description of the BLESS trial (October 2008 to December 2011; Australian New Zealand Clinical Trials Registry ACTRN12608000460303) has been published previously (4). Subjects with non-cystic fibrosis bronchiectasis were aged 20 to 85 years, had a history of 2 or more infective exacerbations in the preceding year, and had been clinically stable (no symptoms of exacerbation or requirement for supplemental antibiotic therapy) for 4 weeks prior to enrollment. Patients were randomized to 48 weeks of twice-daily oral doses of 400 mg erythromycin ethylsuccinate or placebo.

Paired samples were available from 43 and 41 patients from the treatment and placebo groups, respectively. Baseline characteristics of the 84 BLESS subjects for whom samples were available did not differ significantly from those of the 117 subjects of the original BLESS trial (see Table S1 in the supplemental material). The baseline characteristics of the members of the treatment and control subgroups were also comparable between this study and the BLESS trial. Significant intergroup differences were observed only in the use of inhaled short-acting β-agonists (SABA) (58% of subjects receiving erythromycin versus 29% of subjects receiving placebo, *P* = 0.017) in this study (Table 3).

**TABLE 3:** Characteristics of study population^a^

Characteristic	Values		*P* value
	Placebo (*n* = 41)	Erythromycin (*n* = 43)	
Age (yrs), median (IQR)	65 (61–70)	63 (57–67)	0.064
Females, *n* (%)	22 (53)	25 (58)	0.826
Duration of bronchiectasis in yrs, median (IQR)	50 (13–60)	50 (23–60)	0.764
Pulmonary function, mean (SD)			
Prebronchodilator FEV1 (liters)	1.83 (±0.61)	1.87 (±0.62)	0.911
Prebronchodilator FEV1 (% predicted)	71.1 (±18.8)	66.5 (±16.8)	0.336
Postbronchodilator FEV1 (liters)	1.93 (±0.63)	1.97 (±0.65)	0.967
Postbronchodilator FEV1 (% predicted)	75.2 (±19.7)	70.1 (±17.3)	0.261
24-h sputum wt (g), median (IQR)	17.8 (12.1–26)	19.9 (10.9–23.9)	0.610
St. George’s respiratory questionnaire score (total), mean (SD)	37.5 (±15.1)	35.4 (±13.6)	0.618
Leicester cough questionnaire score, mean (SD)	15.2 (±2.86)	15.0 (±2.98)	0.778
6 min walk test (m), median (IQR)	515 (475–575)	512 (487.5–552.5)	0.714
C-reactive protein concentration (mg/liter), median (IQR)	1.9 (0.8–7.3)	3.4 (1.6–9.2)	0.187
Sputum neutrophils (% of nonsquamous cells), median (IQR)	96.0 (91.8–97.1)	97.1 (95.3–98.0)	0.070
Drug treatments, *n* (%)			
Combination (inhaled corticosteroids plus LABA)	13 (31.7)	20 (46.5)	0.169
Inhaled LABA alone	0 (0)	3 (7.3)	0.241
Inhaled SABA alone	12 (29)	24 (58)	0.017^b^
Inhaled corticosteroids alone	5 (12.2)	4 (9.3)	0.735
Prednisolone	3 (7.3)	0 (0)	0.112
Nebulized saline solution	1 (2.4)	0 (0)	0.488
Inhaled mannitol	0 (0)	1 (2.3)	>0.999
Comorbidities, *n* (%)			
Ciliary dysfunction	1 (2.4)	1 (2.3)	>0.999
Hypertension	16 (39.0)	11 (25.6)	0.192
Ischemic heart disease	4 (9.8)	3 (7.0)	0.710
Cerebrovascular disease	4 (9.8)	2 (4.7)	0.427
Diabetes mellitus	1 (2.4)	1 (2/3)	>0.999

a Data represent means ± standard deviations (SD), number (percent), or median (IQR) as indicated. *P* values were calculated using the Mann-Whitney test or Fisher exact test according to the characteristics of the data distribution. FEV1, forced expiratory volume in 1 s. FEV1 (% predicted), FEV1 as a percentage of the predicted value; ICS, inhaled corticosteroid; LABA, long-acting β-agonists; SABA, short-acting β-agonists.

b *P* value of <0.05.

### Sample collection.

Oropharyngeal swabs were collected at baseline and at study completion (trial week 48). Samples were obtained by means of a swab pressed over the tonsils and posterior pharyngeal wall, avoiding jaws, teeth, and gingiva on withdrawal. Sample collection was performed when participants visited the center, and samples were stored in STGG (skim milk, tryptone, glucose, glycerin) medium (43) at −80°C prior to analysis.

### DNA extraction, 16S rRNA gene amplicon sequencing, and bioinformatics processing.

Swabs were subjected to vortex mixing in the collection medium for 30 s, and bacterial cells were pelleted by centrifugation at 13,000 × *g* for 10 min. Cell pellets were subjected to bead beating (silica/zirconium [1:1 ratio of 0.1 and 1.0 µM] and chrome beads) (Daintree Scientific, Tasmania, Australia) with a FastPrep-24 instrument at 6.5 m/s for 1 min, followed by incubation for 1 h at 37°C in 2.9 mg/ml lysozyme and 0.14 mg/ml lysostaphin (Sigma-Aldrich, St. Louis, MO, USA). DNA extraction was performed using a GenElute Bacterial Genomic DNA kit, in accordance with the instructions of the manufacturer (Sigma-Aldrich, St. Louis, MO, USA).

V1-V3 region 16S rRNA gene amplicon sequencing was performed on an Illumina MiSeq platform at the South Australian Health and Medical Research Institute, as described previously (22). Details of library preparation and sequencing are provided in Text S1 in the supplemental material. Sample processing and analysis were performed using a methodology designed for low-biomass contexts (43). Operational taxonomic unit (OTU) assignment was performed using an open reference approach with the UCLUST algorithm based on 97% similarity to the SILVA reference database (version 111). Prior to OTU assignment, sequences with less than 80% similarity to sequences in the reference databases were discarded. Following the removal of spurious operational taxonomic units (OTUs) such as those assigned as mitochondria and chloroplasts, sequence data were subsampled to 6,953 reads, providing an average level of Good’s coverage of 98.3%. OTUs with ≤10 reads across the sample cohort were removed. Two samples from the placebo group and two samples from the erythromycin group failed to reach quality thresholds (specifically, they showed low microbial richness and diversity, suggesting contamination by sputum) and were removed.

10.1128/mSphere.00103-18.1 TEXT S1  Supplemental materials and methods. Download TEXT S1, PDF file, 0.2 MB. © Crown copyright 2018. 2018 Crown This content is distributed under the terms of the Creative Commons Attribution 4.0 International license.

### Multiplex PCR for antibiotic resistance genes.

Carriage of *erm*(A), *erm*(B), *erm*(C), *erm*(F), *msrA*, and *mef* genes was assessed by single or multiplex PCR (Table S2), as described in Text S1. DNA bands were visualized on a 2.5% agarose gel on a GeneGenius bioimaging system (Syngene, Frederick, MD, USA). Assay specificity was confirmed by Sanger sequencing.

10.1128/mSphere.00103-18.7 TABLE S2  Primer sequences and amplicon sizes of antibiotic resistance and positive control (16S rRNA) genes for single or multiplex PCR and quantitative PCR, respectively. Download TABLE S2, PDF file, 0.1 MB. © Crown copyright 2018. 2018 Crown This content is distributed under the terms of the Creative Commons Attribution 4.0 International license.

### Quantitation of bacterial load, resistance gene carriage, and specific bacterial taxa.

A quantitative PCR (qPCR) assay targeting the 16S rRNA gene was used to assess total bacterial load (44). Levels of *erm*(B) (45) and *erm*(F) (46) were assessed with SYBR green assays, and levels of the *mef* gene were assessed with the TaqMan assay (47), using primers described in Table S2. Assessment of the abundances of specific bacterial taxa was performed using SYBR green and TaqMan qPCR assays (Table S3), as detailed in Text S1. Δ*C*_*T*_ values are based on differences in *C*_*T*_ values between the target gene and reference (16S rRNA) gene.

10.1128/mSphere.00103-18.8 TABLE S3  Primer sequences and PCR annealing conditions for quantitative PCR of specific bacterial taxa. Download TABLE S3, PDF file, 0.1 MB. © Crown copyright 2018. 2018 Crown This content is distributed under the terms of the Creative Commons Attribution 4.0 International license.

### Statistical analysis.

Alpha diversity measures (taxon richness [observed species], Simpson’s index [one-dimensional {1-D}], Shannon diversity, and Faith’s phylogenetic diversity) were computed in QIIME (v1.8.0). Multivariate statistical analysis of 16S rRNA gene profiles was performed using primer 6 software (Primer-E Ltd., Plymouth, United Kingdom). Beta diversity was assessed using a Bray-Curtis distance matrix based on standardized data. Sample distances were visualized by nonmetric multidimensional scaling (NMDS) (48). Taxa that were present (prevalence) in at least 90% of the population at a relative abundance of greater than 0.1% were defined as members of the core microbiome of the oropharynx. Fold change in OTU relative abundance between groups and time points was determined using *R* DESEQ2 statistical software (49) within the phyloseq package (50), with the Benjamini-Hochberg false-discovery-rate (FDR) correction for multiple comparisons. Identification of significant OTUs was based on the closest taxonomic assignment as assessed using the Ribosomal Database project (RDP; release 11) based on the sequence match S_ab score (51). Further phylogenetic analysis of 16S rRNA gene sequences was performed using ARB software (52). Phylotypes were added to the SILVA phylogenetic tree using the parsimony method, preserving the overall tree topology, and annotations were performed using interactive tree of life (iTOL) software (53).

### Accession number(s).

Sequencing data were deposited in the public SRA database (accession no. PRJNA379755).
