# Supplementary figures and images for: Impact of Long-Term Erythromycin Therapy on the Oropharyngeal Microbiome and Resistance Gene Reservoir in Non-Cystic Fibrosis Bronchiectasis
Source: mSphere. 2018 Apr 18;3(2):e00103-18. doi: 10.1128/mSphere.00103-18 (PMC5907653; doi:10.1128/mSphere.00103-18)

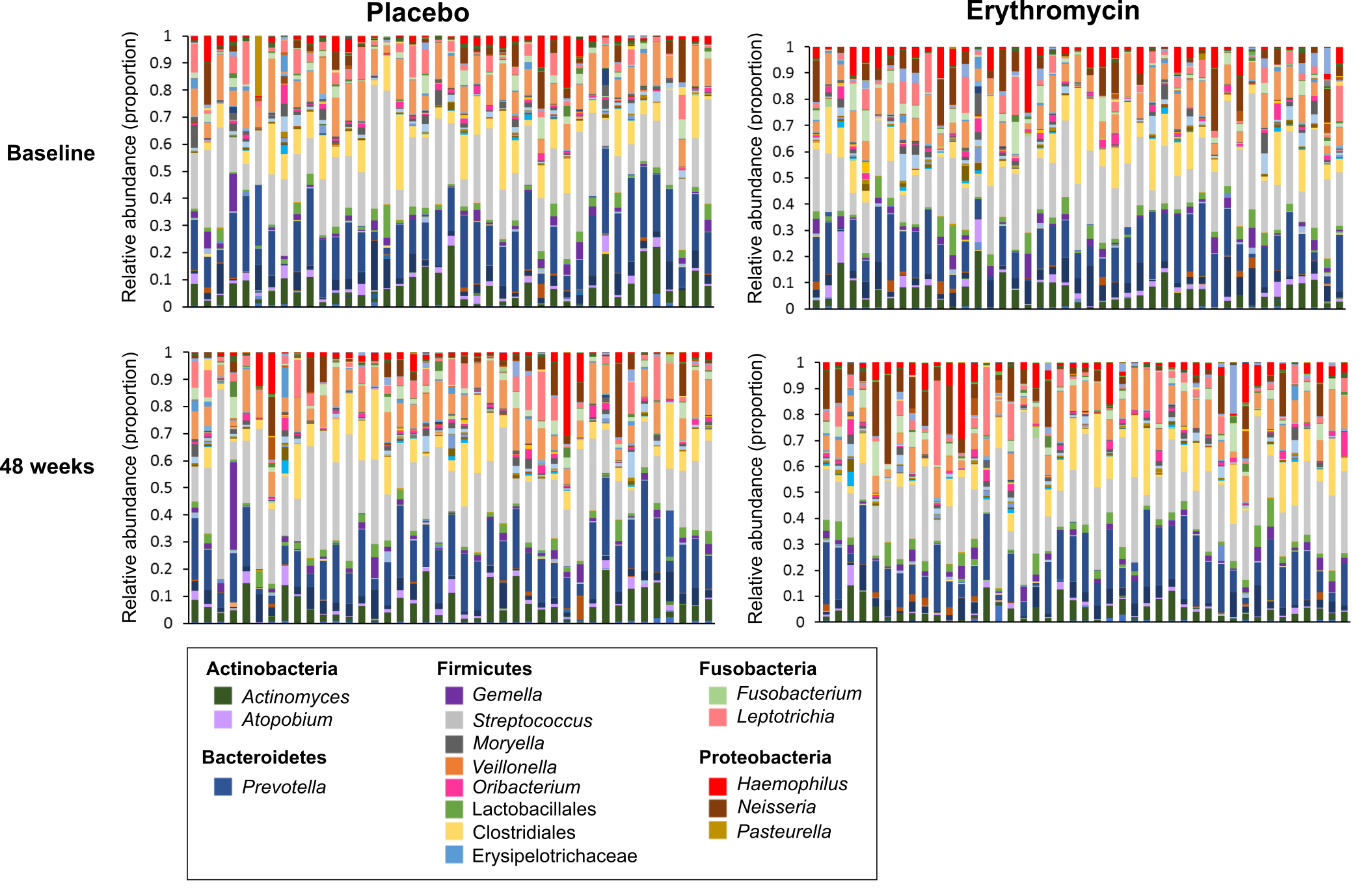

Supplement: FIG S1 [file sph002182523sf1.tif]

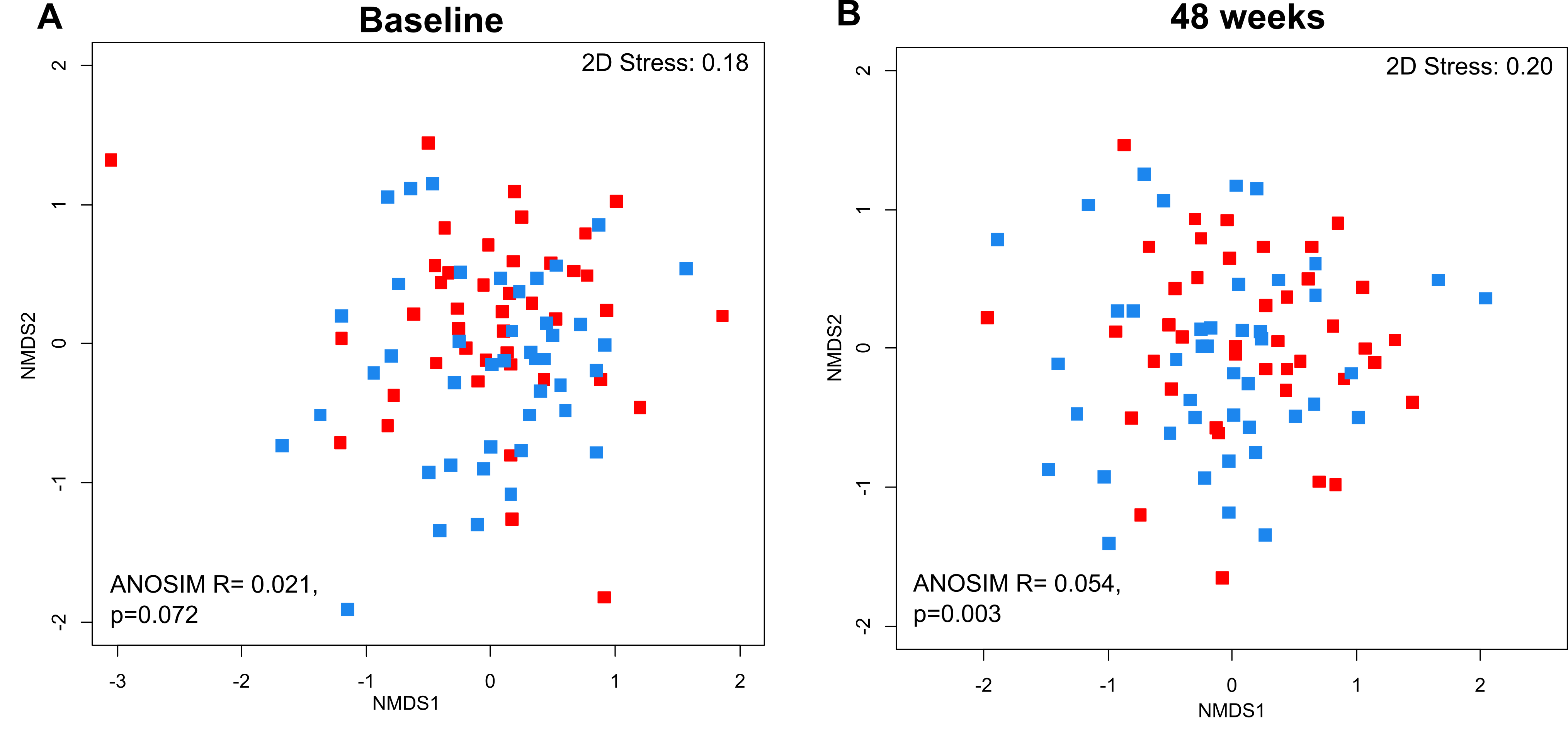

Supplement: FIG S2 [file sph002182523sf2.tif]

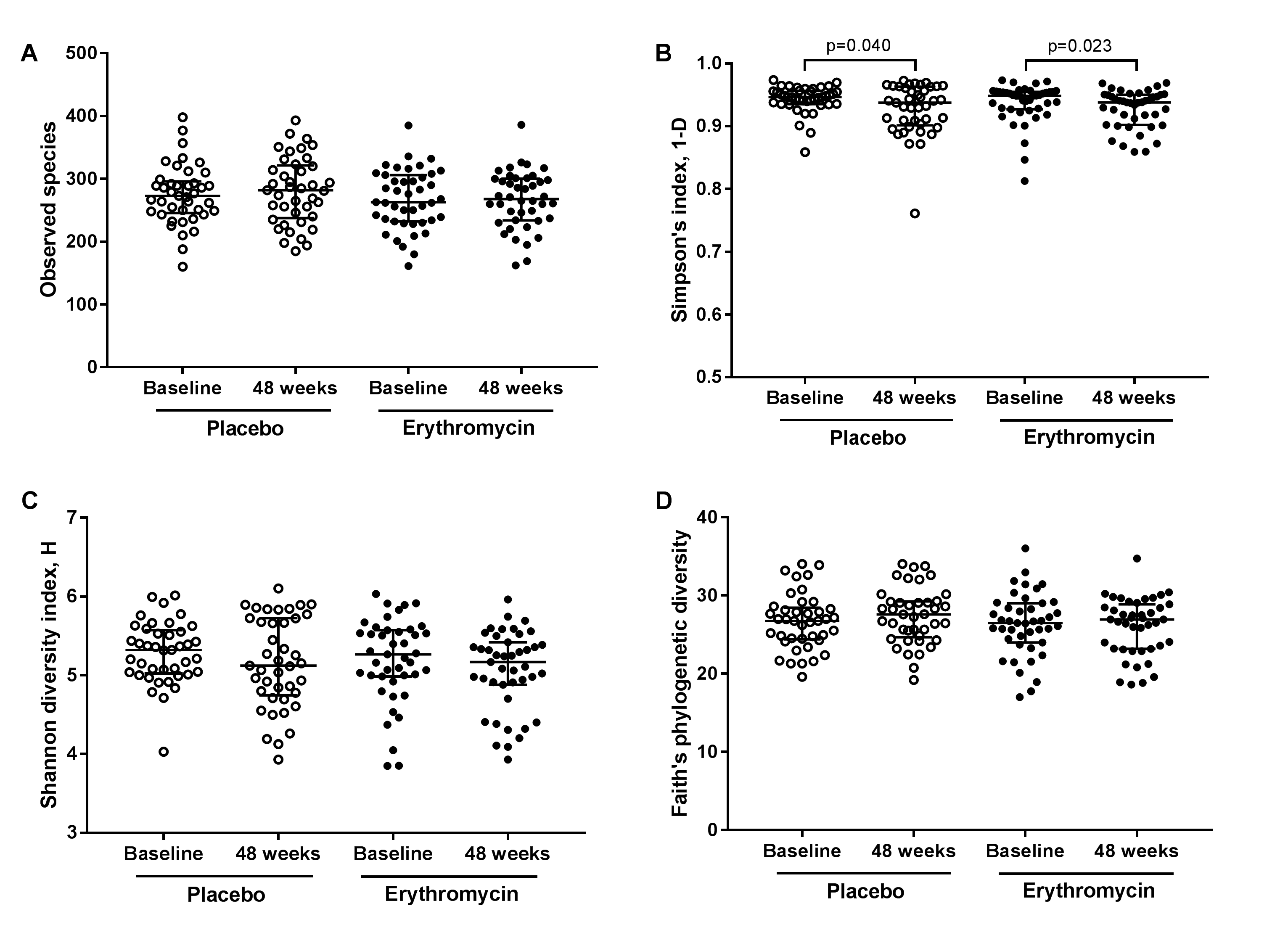

Supplement: FIG S3 [file sph002182523sf3.tif]

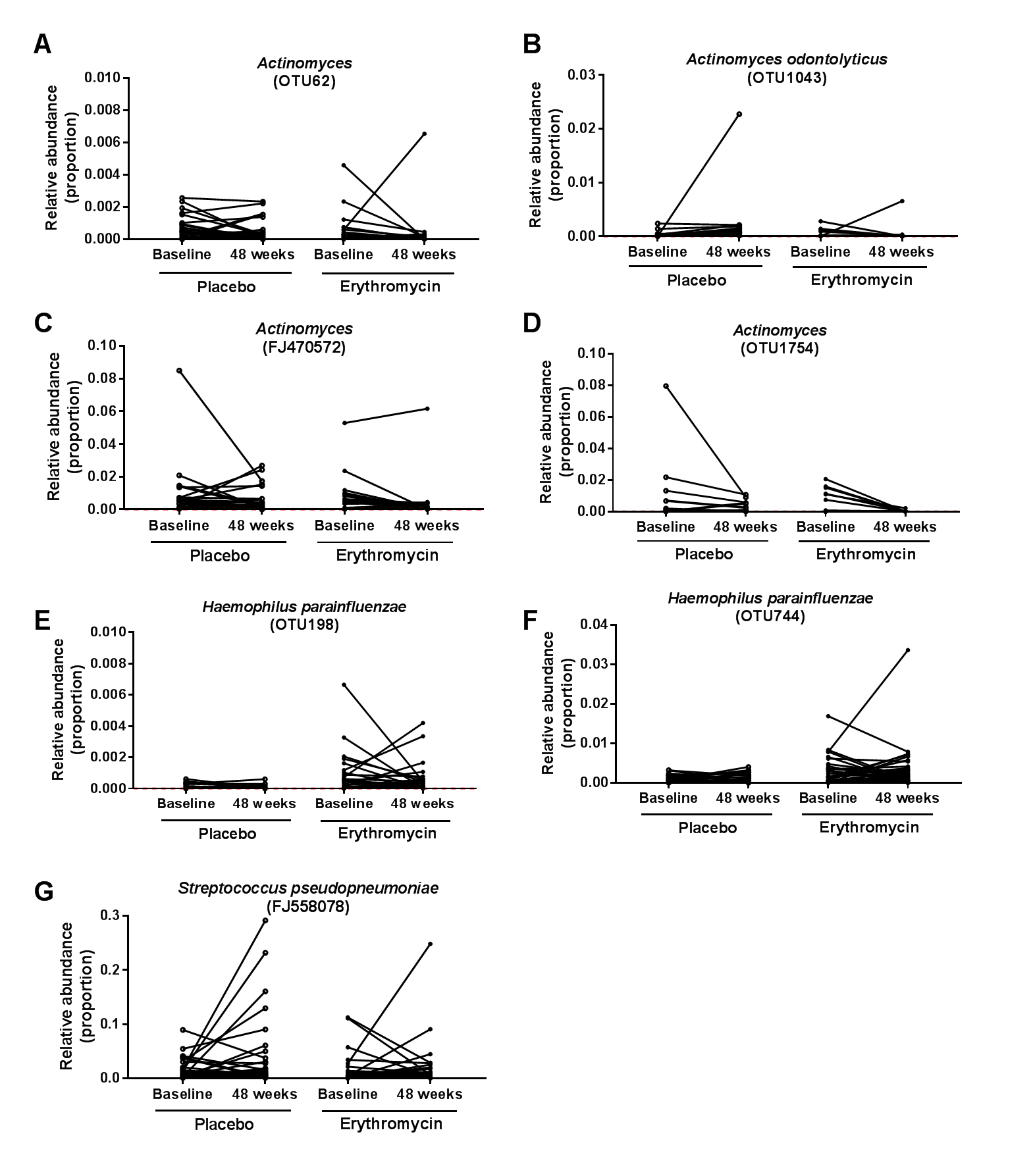

Supplement: FIG S4 [file sph002182523sf4.tif]
