# Supplementary material for: Impact of Long-Term Erythromycin Therapy on the Oropharyngeal Microbiome and Resistance Gene Reservoir in Non-Cystic Fibrosis Bronchiectasis
Source: mSphere. 2018 Apr 18;3(2):e00103-18. doi: 10.1128/mSphere.00103-18 (PMC5907653; doi:10.1128/mSphere.00103-18)
Supplement: TABLE S1 [file sph002182523st1.pdf]

	Current cohort (n= 84)	Original cohort (n=117)	***P*-value**
Age (years), median (IQR)	64 (59-69.25)	64 (59-68)	1
Females, n (%)	47 (56%)	71 (61%)	0.562
Duration of bronchiectasis in years, median (IQR)	50 (20-60)	50 (15-60)	0.489
Pulmonary function, mean (SD)			
Prebronchodilator FEV1 (L)	1.85 (±0.62)	1.82 (±0.71)	0.536
Prebronchodilator FEV1 (% predicted)	68.7 (±17.9)	68.5 (±18.7)	0.993
Postbronchodilator FEV1 (L)	1.95 (±0.64)	1.90 (±0.74)	0.442
Postbronchodilator FEV1 (% predicted)	72.6 (±18.7)	71.9 (±18.9)	0.874
24 hours sputum weight (g), median (IQR)	18.4 (12.0-25.0)	17.1 (10.0-24.5)	0.480
St George's Respiratory Questionnaire score (total), mean (SD)	36.4 (±14.4)	37.1 (±14.5)	0.814
Leicester Cough Questionnaire score, mean (SD)	15.1 (±2.9)	14.9 (±3.2)	0.754
6 min walk test (m), median (IQR)	512 (475-571)	510 (475-570)	0.670
C-reactive protein concentration (mg/L), median (IQR)	3.05 (1.1-8.9)	3.3 (1.1-8.1)	0.950
Sputum neutrophils (% of non-squamous cells), median (IQR)	96.4 (93.5-97.7)	96.3 (93.1-97.5)	0.538
Drug treatments, n (%)			
Combination (inhaled corticosteroids plus LABA)	33 (39.3%)	51 (43.6%)	0.565
Inhaled LABA alone	3 (3.6%)	4 (3.4%)	>0.999
Inhaled SABA alone	36 (42.9%)	49 (41.8%)	>0.999
Inhaled corticosteroids alone	9 (10.7%)	13 (11.1%)	>0.999
Prednisolone	3 (3.6%)	3 (2.6%)	0.697
Nebulised saline	1 (1.2%)	2 (1.7%)	>0.999
Inhaled mannitol	1 (1.2%)	1 (0.9%)	>0.999
Comorbidities, n (%)			
Ciliary dysfunction	2 (2.4%)	3 (2.6%)	>0.999
Hypertension	27 (32.1%)	37 (31.6%)	>0.999
Ischaemic heart disease	7 (8.3%)	11 (9.4%)	>0.999
Cerebrovascular disease	6 (7.1%)	6 (5.1%)	0.562
Diabetes mellitus	2 (2.4%)	3 (2.6%)	>0.999

Bold indicates p-value <0.05. Data are mean ± SD, n (%) or median (IQR). *P*-values are calculated by Mann-Whitney test or Fisher's exact test according to the characteristics of the data distribution. FEV1 (% predicted), FEV1 as a percentage of the predicted value; ICS, inhaled corticosteroid; LABA, long-acting β-agonists; SABA, short-acting β-agonists.
