# Supplementary material for: Impact of Long-Term Erythromycin Therapy on the Oropharyngeal Microbiome and Resistance Gene Reservoir in Non-Cystic Fibrosis Bronchiectasis
Source: mSphere. 2018 Apr 18;3(2):e00103-18. doi: 10.1128/mSphere.00103-18 (PMC5907653; doi:10.1128/mSphere.00103-18)
Supplement: TEXT S1 [file sph002182523s1.pdf]

THE IMPACT OF LONG-TERM ERYTHROMYCIN THERAPY ON OROPHARYNGEAL MICROBIOME AND RESISTANCE GENE RESERVOIR IN NON-CF BRONCHIECTASIS

Jocelyn M. Choo, Guy C.J. Abell, Rachel Thomson, Lucy Morgan, Grant Waterer, David L. Gordon, Steven L. Taylor, Lex E.X. Leong, Steve L. Wesselingh, Lucy D. Burr, Geraint B. Rogers

SUPPLEMENTARY MATERIAL AND METHODS

16S rRNA gene sequencing and bioinformatics processing

16S rRNA amplicon generation and indexing were performed according to the Illumina 16S Metagenomic Sequencing Library Preparation protocol (Illumina Inc., San Diego, USA) with modifications. In brief, amplicons of the V1-V3 hypervariable region were generated using the modified universal primers 27F and 519R, as described previously (S1). Dual-indexing of amplicons were performed using the Nextera XT Index kit (Illumina) to enable multiplexing. Samples were pooled at equal concentration and barcoded libraries were quantified using the Qubit dsDNA HS assay (Life Technologies, Carlsbad, USA). Paired-end sequencing (2 x 300 bp) was performed on an Illumina Miseq platform at the David R Gunn Genomics Facility, South Australian Health and Medical Research Institute. Good's coverage of 98.3% was calculated using QIIME (S2) to ensure sufficient coverage at a subsampled depth of 6,953 reads.

Multiplex PCR for antibiotic resistance genes

Carriage of *erm(A), erm(B), erm(C), erm(F), mef* and *msrA* genes was assessed by multiplex PCR or single gene PCR, using previously described primer pairs (Supplementary Table S2). Multiplex PCR reaction consisted of 1X KAPA buffer, 3.5 mM MgCl2, 0.36 mM dNTPs, 0.75U KAPA Taq DNA polymerase (KAPA Biosystems, Wilmington, USA) and the following concentrations of each forward and reverse primer pair: 0.16 μM for *erm(A),* 0.5 μM for *erm(B),* 0.4 μM for *erm(C),* 0.48 μM for *msrA*, 0.4 μM for *mef* and 0.24 μM for 16S. Similar concentrations were used in single PCR for the *erm(F)* gene, with 0.4 μM of each the forward and reverse primer. A total reaction volume of 12.5 µL was achieved by adding the appropriate volume of sterile water. Positive control sample(s) that contained templates of the genes tested were included in all PCR reactions. The multiplex PCR cycle involves initial denaturation at 93°C for 3 min, followed by 45 cycles of 93°C for 1 min, 62°C for 1.5 min and 68°C for 2 min, and a final elongation step at 68°C for 4 min were performed on a Veriti 96-well Thermal Cycler (Life Technologies, Carlsbad, USA). For the *erm(F)* single gene PCR, a melting temperature of 50°C was used. DNA bands were visualized on a 2.5% agarose gel on a GeneGenius bio-imaging system (Syngene, Frederick, USA). Amplicons of each resistance gene was confirmed for specificity by Sanger sequencing.

Quantitation of total bacterial load, resistance gene carriage and specific bacterial taxa

A quantitative PCR (qPCR) assay targeting the 16S rRNA gene was used to assess total bacterial load, as described previously (S3). Levels of *erm(B)* and *erm(F)* genes were assessed using SYBR Green assays, and *mef* gene was assessed using Taqman assay based on previously described primer pairs (Supplementary Table S2). For SYBR Green qPCR assays, 1 µL of DNA extract, 0.2 µM of each primer, 17.5 µL of 2X Platinum SYBR Green qPCR SuperMix-

UDG (Invitrogen, Carlsbad, USA) and the appropriate volume of water was added to a 35 µL total reaction volume. For Taqman qPCR assays, 1 µL of DNA extract, 0.2 µM of each primer, 0.1 µM of target probe, 17.5 µL of 2X KAPA Probe Fast qPCR Master Mix (KAPA Biosystems Inc., Wilmington, USA) and the appropriate volume of water was added to a 35 µL total reaction volume. Quantitative RT-PCR were performed on three technical replicates, at 10 µL reaction volume per replicate, on a QuantStudio 6 and 7 Flex Real-Time PCR system (Applied Biosystems, Carlsbad, USA). Cycling conditions for SYBR Green qPCR assays were: 50°C for 2 min, 95°C for 10 min, followed by 40 cycles of 95°C for 15 secs and 60°C for 1 min. Melt curve analysis was then performed at the following conditions: 95°C for 15 secs, followed by an initial stage temperature of 60°C for 1 min and a final temperature of 95°C for 15 secs, with readings recorded at increments of 0.05°C/s. Cycling conditions for Taqman qPCR assay were: 50°C for 2 min, 95°C for 10 min, followed by 40 cycles of 95°C for 15 secs and 62°C for 1 min. Standard curves were generated for each qPCR reaction based on serial dilutions of *Escherichia coli* genomic DNA for the 16S rRNA gene, *Enterococcus faecalis* genomic DNA for the *erm(B)* gene, as well as a 0.82 kb *erm(F)* and a 1.2 kb *mef* purified PCR template.

Quantitation of *Actinomyces spp.*, *A. odontolyticus* and *S. pneumoniae* was performed using the SYBR Green qPCR assays, while *H. parainfluenzae, H. influenzae* and *S. pseudopneumoniae* was quantified using the Taqman qPCR assay, as described above with modifications. Quantitation of *S. pneumoniae/pseudopneumoniae* was performed using a primer only assay, while the addition of a probe was used to quantify *S. pseudopneumoniae*, as previously described (S4). Modifications to the qPCR reaction include the annealing conditions and also primer concentrations at 0.5 µM were used for *H. influenzae* and *H. parainfluenzae* qPCR assays (Supplementary Table S3).

References

S1. **Taylor SL, Leong LEX, Choo JM, Wesselingh S, Yang IA, Upham JW, Reynolds PN, Hodge S, James AL, Jenkins C, Peters MJ, Baraket M, Marks GB, Gibson PG, Simpson JL, Rogers GB.** 2017. Inflammatory phenotypes in severe asthma are associated with distinct airway microbiology. J Allergy Clin Immunol doi:10.1016/j.jaci.2017.03.044.

S2. **Good IJ.** 1953. The population frequencies of species and the estimation of population of parameters. Biometrika **40:**237-264.

S3. **Nadkarni MA, Martin FE, Jacques NA, Hunter N.** 2002. Determination of bacterial load by real-time PCR using a broad-range (universal) probe and primers set. Microbiology **148:**257-266.

S4. **Sistek V, Boissinot M, Boudreau DK, Huletsky A, Picard FJ, Bergeron MG.** 2012. Development of a real-time PCR assay for the specific detection and identification of *Streptococcus pseudopneumoniae* using the *recA* gene. Clin Microbiol Infect **18:**1089-1096.
