# Supplementary material for: Impact of Long-Term Erythromycin Therapy on the Oropharyngeal Microbiome and Resistance Gene Reservoir in Non-Cystic Fibrosis Bronchiectasis
Source: mSphere. 2018 Apr 18;3(2):e00103-18. doi: 10.1128/mSphere.00103-18 (PMC5907653; doi:10.1128/mSphere.00103-18)
Supplement: TABLE S2 [file sph002182523st2.pdf]

Target gene	Sequence (5' - 3')	Amplicon size (bp)	References
**Single or multiplex PCR**			
erm(A)	5'-TCAGTTACTGCTATAGAAATTGATGGAG-3'<br>5'-ATACAGAGTCTACACTTGGCTTAGG-3'	358	(S1)
erm(B)	5'-TGGTATTCCAAATGCGTAATG-3'<br>5'-CTGTGGTATGGCGGGTAAGT-3'	745	(S2)
erm(C)	5'-CTTGTTGATCACGATAATTTCC-3'<br>5'-ATCTTTTAGCAAACCCGTATTC-3'	190	(S3)
erm(F)	5'-CGGGTCAGCACTTTACTATTG-3'<br>5'-GGACCTACCTCATAGACAAG-3'	466	(S4)
msr(A)	5'-TCCAATCATTGCACAAAATCTAAC-3'<br>5'-AAGTTATATCATGAATAGATTGTCCTGTT-3'	939	(S3)<br>(S5)
mef(A/E)	5'-GATCYGCGATGGTCTTG-3'<br>5'-AAGCTGTTCCAATGCTACGG-3'	229	(S6)<br>(S2)
16S	5'-TCCTACGGGAGGCAGCAGT-3'<br>5'-GGACTACCAGGGTATCTAATCCTGTT-3'	466	(S7)
**Quantitative PCR**			
erm(B)	5'-GAAAGCCRTGCGTCTGACATC-3'<br>5'-CGAGACTTGAGTGTGCAAGAGC-3'	105	(S8)
erm(F)	5'-CGGGTCAGCACTTTACTATTG-3'<br>5'-GGACCTACCTCATAGACAAG-3'	466	(S4)
mef	5'-TATGGAGCTACCTGTCTGGA-3'<br>5'-GGTACTAAAAGTGGCGTAACC-3'<br>HEX-CCGTAGCATTGGAACAGCTTTTC-BHQ1 [Probe]	85	(S9)

References

S1. **Jung JH, Yoon EJ, Choi EC, Choi SS.** 2009. Development of TaqMan probe-based real-time PCR method for *erm(A),erm(B),* and *erm(C),* rapid detection of macrolide-lincosamide-streptogramin B resistance genes, from clinical isolates. J Microbiol Biotechnol **19:**1464-1469.

S2. **Malhotra-Kumar S, Lammens C, Piessens J, Goossens H.** 2005. Multiplex PCR for simultaneous detection of macrolide and tetracycline resistance determinants in streptococci. Antimicrob Agents Chemother **49:**4798-4800.

S3. **Martineau F, Picard FJ, Lansac N, Menard C, Roy PH, Ouellette M, Bergeron MG.** 2000. Correlation between the resistance genotype determined by multiplex PCR assays and the antibiotic susceptibility patterns of *Staphylococcus aureus* and Staphylococcus epidermidis. Antimicrob Agents Chemother **44:**231-238.

S4. **Chung WO, Werckenthin C, Schwarz S, Roberts MC.** 1999. Host range of the ermF rRNA methylase gene in bacteria of human and animal origin. J Antimicrob Chemother **43:**5-14.

S5. **Lina G, Quaglia A, Reverdy ME, Leclercq R, Vandenesch F, Etienne J.** 1999. Distribution of genes encoding resistance to macrolides, lincosamides, and streptogramins among staphylococci. Antimicrob Agents Chemother **43:**1062-1066.

S6. **Klomberg DM, de Valk HA, Mouton JW, Klaassen CH.** 2005. Rapid and reliable real-time PCR assay for detection of the macrolide efflux gene and subsequent discrimination between its distinct subclasses *mef(A)* and *mef(E)*. J Microbiol Methods **60:**269-273.

S7. **Nadkarni MA, Martin FE, Jacques NA, Hunter N.** 2002. Determination of bacterial load by real-time PCR using a broad-range (universal) probe and primers set. Microbiology **148:**257-266.

S8. **Zhang L, Kinkelaar D, Huang Y, Li Y, Li X, Wang HH.** 2011. Acquired antibiotic resistance: are we born with it? Appl Environ Microbiol **77:**7134-7141.

S9. **Srinivasan V, du Plessis M, Beall BW, McGee L.** 2011. Quadriplex real-time polymerase chain reaction (*lytA, mef, erm, pbp2b(wt)*) for pneumococcal detection and assessment of antibiotic susceptibility. Diagn Microbiol Infect Dis **71:**453-456.
