# Supplementary material for: Impact of Long-Term Erythromycin Therapy on the Oropharyngeal Microbiome and Resistance Gene Reservoir in Non-Cystic Fibrosis Bronchiectasis
Source: mSphere. 2018 Apr 18;3(2):e00103-18. doi: 10.1128/mSphere.00103-18 (PMC5907653; doi:10.1128/mSphere.00103-18)
Supplement: TABLE S3 [file sph002182523st3.pdf]

Organism	Sequence (5' - 3')	Annealing conditions (temperature, extension time)	References
Actinomyces spp.	5'-GGCKTGCGGTGGGTACGGGC-3'<br>5'-GGCTTTAAGGGATTCGCTCCRCCTCAC-3'	66°C, 60 s	(S1)
Actinomyces odontolyticus	5'-CTTTGGGATAACGCCGGGAAAC-3'<br>5'-CTACCCGTCAAAGCCTTGGT-3'	60°C, 60 s	(S2)
Haemophilus parainfluenzae	5'-ACGTGGTTTGCGATCCGACGC-3'<br>5'-CCGATTTTGATCTTTGCCCAAGGG-3'<br>[Probe]<br>FAM-TCAAGCCGTGATGGTTTTACCATTGTTG-TAMRA	58°C, 45 s	This study
Haemophilus influenzae	5'-ATTAAATGTTGCATCAACGC-3'<br>5'-GACTTTTGCCCACGCAC-3'<br>[Probe]<br>FAM-ACGRTTTTACCATAGTTGCACTTTCTC-BHQ1	58°C, 45 s	(S3)
Streptococcus pneumoniae/<br>*Streptococcus pseudopneumoniae*	5'-GTGCTITGAAATTCTATGCTTC-3'<br>5'-GTGGAGCTACCTTATTTTTAC-3'<br>[Probe for *S. pseudopneumoniae*]<br>TGGTA**ACA**CA**C**AAATTAAG	60°C, 15 s	(S4)

Residues in bold and underlined are locked nucleotides.

References

S1. **Xia T, Baumgartner JC.** 2003. Occurrence of *Actinomyces* in infections of endodontic origin. J Endod **29:**549-552.

S2. **Yang R, Zou J, Li JY.** 2007. Study of the relationship between oral *Actinomyces* and childhood caries. Hua Xi Kou Qiang Yi Xue Za Zhi **25:**568-570.

S3. **Reddington K, Schwenk S, Tuite N, Platt G, Davar D, Coughlan H, Personne Y, Gant V, Enne VI, Zumla A, Barry T.** 2015. Comparison of Established Diagnostic Methodologies and a Novel Bacterial smpB Real-Time PCR Assay for Specific Detection of *Haemophilus influenzae* Isolates Associated with Respiratory Tract Infections. J Clin Microbiol **53:**2854-2860.

S4. **Sistek V, Boissinot M, Boudreau DK, Huletsky A, Picard FJ, Bergeron MG.** 2012. Development of a real-time PCR assay for the specific detection and identification of *Streptococcus pseudopneumoniae* using the *recA* gene. Clin Microbiol Infect **18:**1089-1096.
